# Heterocyclic aromatic amines in doner kebab: Quantitation using an efficient microextraction technique coupled with reversed‐phase high‐performance liquid chromatography

**DOI:** 10.1002/fsn3.1262

**Published:** 2019-11-28

**Authors:** Abdorreza Mohammadi, Fatemeh Barzegar, Marzieh Kamankesh, Amin Mousavi Khaneghah

**Affiliations:** ^1^ Department of Food Science and Technology Faculty of Nutrition Science Food Science and Technology/National Nutrition and Food Technology Research Institute Shahid Beheshti University of Medical Sciences Tehran Iran; ^2^ Food Safety Research Center Shahid Beheshty University of Medical Sciences Tehran Iran; ^3^ Department of Food Science Faculty of Food Engineering University of Campinas (UNICAMP) Campinas São Paulo Brazil

**Keywords:** central composite design, doner kebab, Heterocyclic aromatic amines, instrumental analysis, meat product, microextraction

## Abstract

The safety of doner kebab as a traditional Middle East tasty food can threaten via the formation of dangerous compounds such as heterocyclic aromatic amines during heat process. In this regard, the current investigation was devoted to measuring of 4 HAAs (2‐amino‐3,4‐dimethylimidazo[4,5‐f]quinoline (MeIQ), 2‐amino‐3,8‐dimethylimidazo[4,5‐f]quinoxaline (MeIQx), 2‐amino‐1‐methyl‐6‐phenylimidazo[4,5‐b]pyridine (PhIP), and 2‐amino‐3‐methylimidazo[4,5‐f]quinoline (IQ)) in doner kebab samples with an innovative microextraction technique combined with high‐performance liquid chromatography. The limit of detection was in the range of 4.8 and 5.3 ng/g, while relative standard deviations were between 6.5% and 8.3%, and recoveries were calculated in the range of 89%–97%. The most and the least total mean values of HAA levels were 13.30 ng/g for MeIQx and 5.0 ng/g for IQ. The proposed method showed a high capability to extract trace amount of HAAs from a complex matrix such as doner kebab. Also, this technique is easy, high sensitive, selective, accurate and efficient.

## Graphical abstract

Current investigation has been devoted to determination of 4 HAAs (2‐amino‐3,4‐dimethylimidazo[4,5‐f]quinoline (MeIQ), 2‐amino‐3,8‐dimethylimidazo[4,5‐f]quinoxaline (MeIQx), 2‐amino‐1‐methyl‐6‐phenyl‐imidazo[4,5‐b]pyridine (PhIP) and 2‐amino‐3‐methylimidazo[4,5‐f]quinoline (IQ)) in five samples of doner kebab with an innovative microextraction technique combined with high‐performance liquid chromatography.

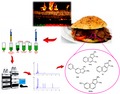

## 1 INTRODUCTION

Animal‐based meat and meat products have been commonly consumed as an important source of protein, vitamin B_12_, iron, folic acid, and minerals in most of the countries around the world (Higgs, 2000; Valsta, Tapanainen, & Männistö, 2005). Due to recent changes in lifestyle, people are less inclined to spend much time on cooking and mostly prefer tasty food diet.

Doner kebab, which is known by other names, such as donair, gyro chawarma, and doner kebab as a traditional Middle East food is prepared by cooking the meat, has become very popular throughout the world (Hosseini et al., 2013; Özsaraç, Kolsarici, Demirok Soncu, & Haskaraca, 2019). It has commonly made with beef, veal, lamb, or even poultry meat (Hosseini et al., 2013). In order to prepare doner kebab, meat is minced with tallow, marinated, and then seasoned with onion, tomatoes, white and black pepper, allspice, cumin, and thyme (Kayaardi, Kundakci, Kayacier, & Gok, 2006; Vazgecer, Ulu, & Oztan, 2004). The mixture is formed like a cone and then doner was spite‐grilled during slow rotation (Kayaardi et al., 2006). A heating process which is typically applied for cooking resulted in the production of pleasant odor and flavor and also toxic compounds such as heterocyclic aromatic amines (HAAs) (Gross et al., 1993; Özsaraç et al., 2019).

**Figure 1:**
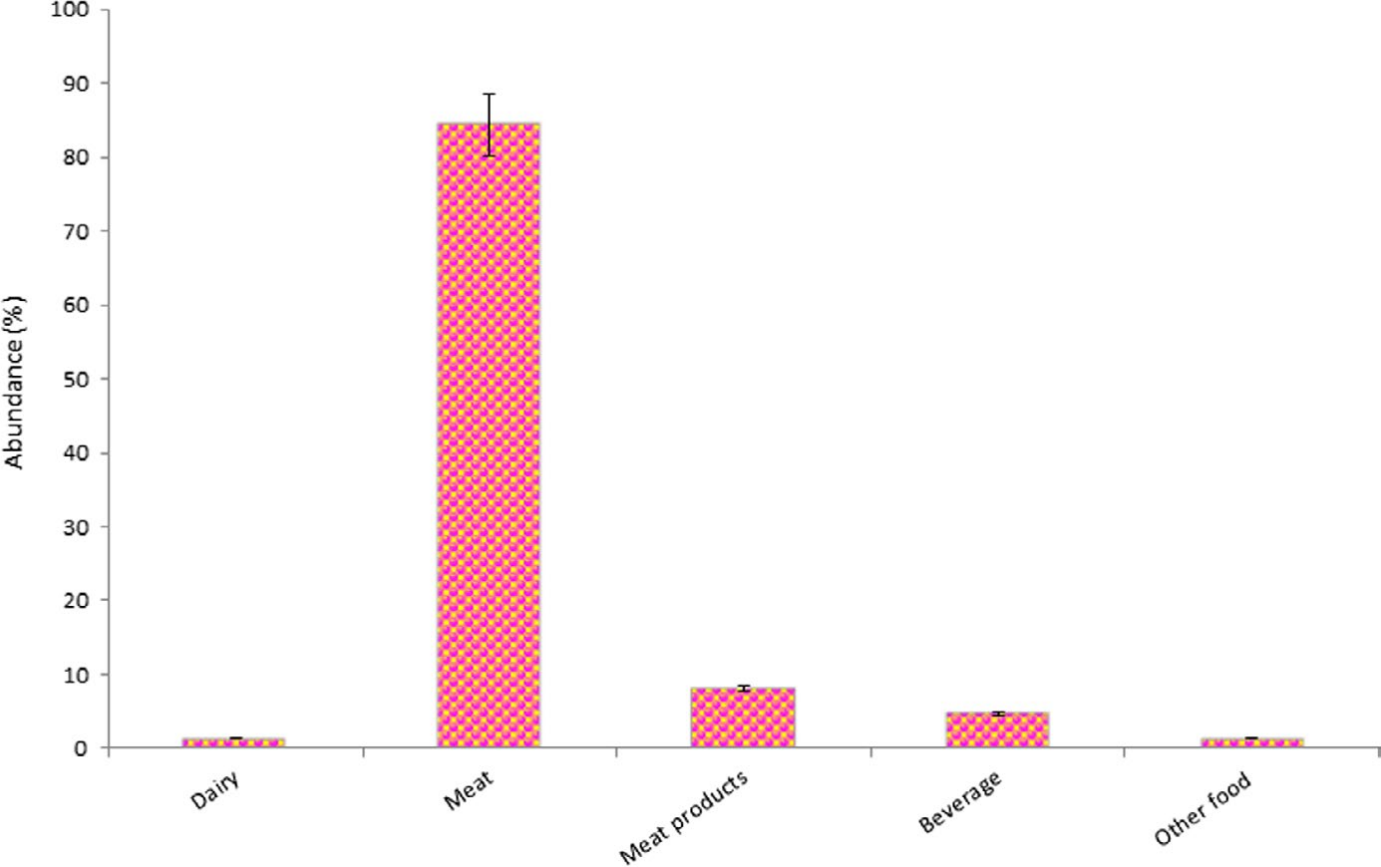
High concentration of highly risk HAAs including PhIP, IQ, MeIQ, and MeIQx in different groups of food

HAAs are known as carcinogenic and mutagenic compounds which are produced via cooking the protein‐rich food in high temperature (Agudelo Mesa, Padró, & Reta, 2013; Casal, Mendes, Fernandes, Oliveira, & Ferreira, 2004). The carcinogenicity of these deleterious compounds is ten times higher than aflatoxins B_1_, nitrosamines, and benzo[*α*]pyrene (Püssa, 2013). The trace level of HAAs in heated meat‐based food can seriously cause illness such as different cancers, for example, prostate, colon, breast, pancreatic, and stomach in the human body (Aeenehvand et al., 2016; Namiranian, Moradi‐Lakeh, Razavi‐Ratki, Doayie, & Nojomi, 2014; Oba et al., 2006; Paluszkiewicz, Smolińska, Dębińska, & Turski, 2012; Puangsombat, Gadgil, Houser, Hunt, & Smith, 2011). Some precursors such as hexoses, free amino acids, creatine, and creatinine are needed for the formation of these toxicants (Zaidi, Kumar, & Rawat, 2012). These chemicals are categorized into two different groups (Yousefi, Shemshadi *et al*., 2018). The first group is called "pyrolytic HAAs," which are formed due to the decomposition of amino acids during pyrolysis at high temperature. The latter one is aminoimidazoarenes (AIAs), which may be formed by cooking the meat under 300ºC. Formation of HAAs is related to some fundamental factors such as temperature and duration of the cooking, type of cooking procedure, meat type, the existence of antioxidants, free amino acids, lipid content, amount of sugar, level of creatine, creatinine, and water activity (Dundar, Sarıçoban, & Yılmaz, 2012). Among 30 types of HAAs, 4 types of these toxicants including MeIQ, MeIQx, PhIP, and IQ have been categorized as high‐risk compounds in which their ingestion can result in the unexpected synthesis of DNA and following that cancer (Puangsombat et al., 2011). According to Figure 1, the formation of these four mentioned HAAs is relatively higher in meat and partially in meat products among the other groups of food products (Barzegar, Kamankesh, & Mohammadi, 2019).

Various analytical techniques have been introduced to quantify of HAAs in food products such as liquid chromatography/fluorescence detection (LC/FLU) (Jautz & Morlock, 2007), gas chromatography/mass spectrometry (GC/MS) (Krach & Sontag, 2000), liquid chromatography/ultraviolet detection (LC/UV) (Agudelo Mesa et al., 2013) and capillary liquid chromatography/mass spectrometry (Gonzalo‐Lumbreras, Rosales‐Conrado, León‐González, Pérez‐Arribas, & Polo‐Díez, 2010), liquid chromatography–mass spectrometry (LC/MS) (Khan, Busquets, Santos, & Puignou, 2008), ultra/liquid chromatography (ULC) (Oz, 2011), and also, LC joined to photo‐diode array detection (HPLC‐DAD) (Dundar et al., 2012).

In another hand, due to the contamination of food samples by trace amounts of HAAs, their designation straightly from solid texture is not conceivable. Hence, sensitive and selective techniques are required for the primary extraction (Barceló‐Barrachina, Santos, Puignou, & Galceran, 2005). Recently, microwave digestion is capable of separating organic components from different textures such as food matrices (Ghasemzadeh‐Mohammadi, Mohammadi, Hashemi, Khaksar, & Haratian, 2012). Some advantages, such as acceptable recovery, using a lower level of organic solvents, decrease extracting time associated with microwave‐assisted extraction (MAE) (Ballard, Mallikarjunan, Zhou, & O'Keefe, 2010).

Due to some disadvantages, such as long extraction time, expensive, high risk of analyte loss, and also low recovery, the application of the traditional methods including, solid‐phase extraction (SPE) (Galceran, Pais, & Puignou, 1996) and liquid–liquid extraction (LLE) (Gu et al., 2002), was combined with some difficulties (Aeenehvand et al., 2016). In this regard, an innovative and efficient sample pretreatment, commonly known as dispersive liquid–liquid microextraction (DLLME), was introduced in 2006 (Rezaee et al., 2006). This technique compensates mentioned shortcomings of classical extraction procedures and offers various benefits such as high adaptability with different analytical instruments, low amount of organic solvents, and to be an operator‐friendly technique which can extract low amount of analytes from complex tissue of foodstuffs with high recovery and also short time (Aeenehvand et al., 2016).

Due to increasing trends in production as well as consumption of doner kebab, the monitoring, extracting, and further determination of possible toxic contaminants, that is, four important including PhIP, MeIQ, MeIQx, and IQ, is crucial. Microextraction process as a fast, sensitive, selective, and safe technique was employed to enrich most target analytes with high recovery from intricate matrices of doner kebab samples. Then, HPLC was applied to quantify HAAs in samples. Optimization step was done using CCD, and the optimal condition was used to determine HAAs in real samples.

## 2 MATERIALS AND METHODS

### 2.1 Chemical reagents

Four kinds of HAA standards including 2‐amino‐3‚4‐dimethlylimidazo [4–5‐f] quinoline (MeIQ), 2‐amino‐3‐methylimidazo [4–5‐f] quinoline (IQ), 2‐amino‐1‐methyl‐6‐phenylimidazo [4–5‐b] pyridine (PhIP), and 2‐amino‐3,8‐dimethylimidazo [4–5‐f] quinoxaline (MeIQx) were obtained from Santa‐Cruz Biotechnology. The stock solution of 1,000 mg/L of these mentioned HAAs has been separately prepared, and also, the mixed stock solutions of standards mentioned above (1–1000 ng/g) were prepared by using methanol. Chemical reagents used in the present study including potassium hydroxide, ethanol, sodium acetate, acetic acid for providing acetate buffer (pH = 3), hydrochloric acid with purity 37% (w/w), 1‐octanol, sodium chloride (NaCl), acetonitrile, and methanol all in analytical grade were purchased from Merck (Darmstadt, Germany). Potassium hexaferrocyanide; carrez I, and zinc acetate; carrez II, was purchased from Panreac (Spain) and Merck, respectively. To prepare carrez I (0.25 mol/L), amount of 10.6 g potassium hexaferrocyanide has been added to 100 ml distilled water. Carrez II (0.4 mol/L) has also been made from 21.9 g zinc acetate and 3 ml acetic acid and 97 ml with distilled water. The concentration of KOH was 1 mol/L.

### 2.2 Instrumentation

HPLC instrument (Agilent 1260 series) as a strong analysis instrument equipped by multiple solvent transfer part, mixing chamber, two powerful CE‐4100 pumps, vacuum degasser, CE‐4200 UV–vis detector (Cambridge, UK), and 6 port valve (Rheodyne). Isolation of HAAs has been done by C_18_ column (250 mm length, 4 mm internal diameter, 5 μm particle size). Due to optimal isolation, acetonitrile and buffer (sodium acetate: 0.2 mol/L, pH = 3) with the ratio of 8:92 (v/v) and suitable flow rate (1 ml/min) applied for optimal separation. The temperature of the column was set at 25°C, and the volume of the injection port was 20 µl. The proper wavelength for HAAs monitoring was 264 nm. The microwave experiment was carried out using microwave digestion (MDS‐10 Sineo).

### 2.3 Sample pretreatment procedure

Five doner kebab samples were purchased from various restaurants through Tehran Province which are cooked for 14 min each side at 200°C and minced using kitchen mixer for achieving a homogeneous sample. One gram of spiked sample (100 ng/g) was mixed with 10 ml of KOH/acetonitrile/ethanol (70:20:10) in the glass container and then microwaved at 520 W for 1 min. After cooling, all contents of the glass container were placed in a new conical falcon and then were centrifuged at 6,037.2 g for 10 min in 25°C. The pH of the solution was adjusted to 3 by adding hydrochloric acid. One mL from each carrez I and carrez II was added into the solution and shaken to precipitate the individual interferences. This solution was centrifuged again at 6,037.2 g for 10 min. The pH of the supernatant phase was adjusted by adding hydroxide potassium (5 mol/L) to 11. In the final step, the solution was centrifuged at 6,037.2 g for 10 min, and the upper clear phase will be used for the next step.

### 2.4 Dispersive liquid–liquid microextraction

In the first step, 10% of NaCl has been added into the solution and was shaken properly. About 650 ml of methanol (dispersing solvent) and 100 ml of 1‐octanol (extracting solvent) were added into the solution and were thoroughly shaken to reach a cloudy solution. HAAs floated in upper phase after centrifuging at 6,037.2 g for 5 min, and then, this phase was separated and injected into HPLC by microsyringe.

### 2.5 Design of experiment

Six important factors including volume and type of both dispersive and extraction solvents, pH level, and polarity of solvent (effect of salt) have been defined in this study to optimize the DLLME technique. According to previous optimization researches, the central composite design (CCD) as the best way of response surface methodology (RSM) has been applied to achieve the best performance (Gomes et al., 2013). Four limits chose for each parameter: the volume of extracting solvent (A): 60–150 µl; volume of dispersive solvent (B): 300–1000 µl; salt (C): 0%–20%; and pH (D): 2–11. Thirty trials with six central points have been achieved using the software package Design‐Expert 8.0.5.

## 3 RESULTS AND DISCUSSIONS

### 3.1 Optimization of main parameters in microextraction

In this trial, the major variables with the highest impacts on extraction efficiency have been optimized. Basic factors such as the potency of analyte extraction, low solubility in water, and proper chromatographic manner should be considered in the selection of extracting solvent. High‐density solvents (HDS) such as chlorinated solvents are incompatible with the mobile phase of HPLC and also have nonpolar property as well. Therefore, 1‐octanol as low‐density solvent (LDS) with lower toxicity than HDS and maximum HAAs extraction yield was chosen as a suitable extraction solvent (Kamankesh, Mohammadi, Hosseini, & Modarres Tehrani, 2015). Methanol among the other solvents such as acetone, ethanol, and acetonitrile was shown a high recovery in extraction procedure and was selected as a proper dispersive solvent. Methanol could prepare the nice cloudy state in the dispersing process. Ethanol has lower cloudy state than methanol and acetone, and acetonitrile could cause a little bit cloudy situation. Also, the extraction solvent level, pH, polarity, and disperser solvent level (salt effect) were optimized, and interaction among these parameters was also surveyed.

ANOVA and regression analysis have been applied to investigate the affecting of parameters. The arranged model offered *R* ^2^ higher than 0.9046, which revealed that the experimental data have an appropriate adaptation with the offered model and also offered model has sufficient ability for prediction. Adjusted *R* ^2^ 0.8063 is high enough to prove the sufficiency of the suggested model. Most obtained points were distributed closely around the linear line from the predictive curve, and this situation confirmed the suitable fit of the presented quadratic model. Also, the normality of the residuals and predicted was corroborated that errors were normally scattered and no contrast of presumption was observed.

**Figure 2:**
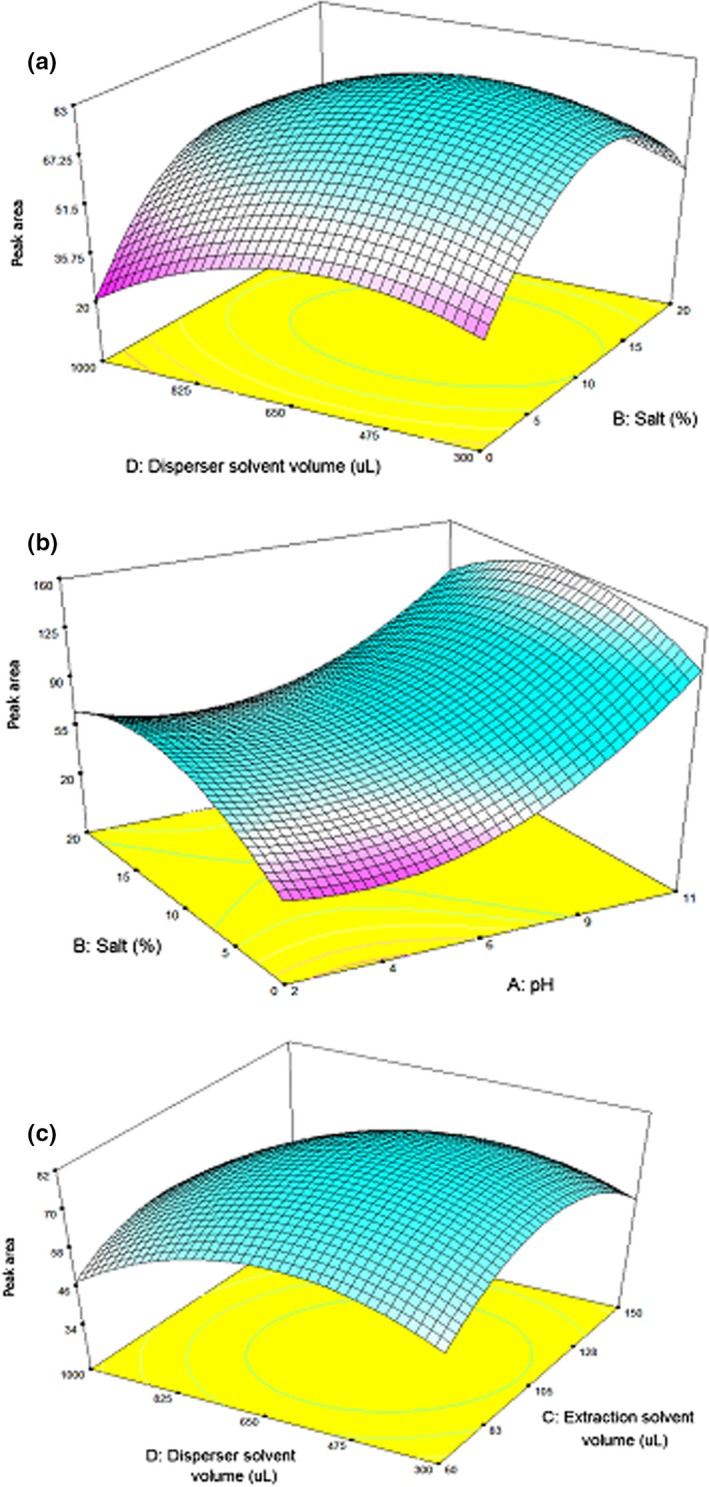
Responses using the central composite design achieved from 3D plotting: (a) salt versus disperser solvent volume, (b) pH versus salt, and (c) extraction solvent volume versus disperser solvent volume

The interaction effects of two parameters on response were indicated in dimensional graphs. Figure 2a presents a significant positive interaction between NaCl and methanol. Totally, because of aggregation of Na^+^ and Cl^‐^ around H_2_O molecules in the sample solution, HAAs could freely move toward extraction solvent, and the salting‐out effect has been observed. In this regard, 10% of NaCl and 650 μl of methanol were shown the optimum values to reach the highest extraction efficiency. The interaction between pH level and salt percent is demonstrated in Figure 2b; the maximum response was achieved by the incorporation of 10% of NaCl and pH 11. As shown in the graph, the response was enhancing up to 10% of NaCl. For salt amount higher than 10 percent, the response was decreasing because molecules of salt could not be properly dissolved in the sample solution in a saturated state. As shown in Figure 2b, the response was linearly increased following by enhancing pH from 2 to 11. In alkaline pH, the concentration of H^+^ is decreased, and the ion type of HAAs is disappeared. On the other hand, HAAs have been deprotonated, and the neutral form of the HAAs has been created. Therefore, the maximum responses occur in pH 11. The highest efficiency related to the interaction between disperser and extracting solvents was achieved at 650 μl of methanol and 100 μl of 1‐octanol (Figure 2c). The volume lower than 100 μl did not show the proper extraction recovery because it did not form a good cloudy state, and for the volume higher than 100 μl, enrichment factor decreased due to the diluted event and the extraction yield was not acceptable. For the volumes lower than 650 μL of methanol did not apear cloudy solution and the extraction of target analyte was done in low recovery. The dilution effect happened for dispersed volume higher than 650 μl of methanol. Therefore, 10% NaCl, 650 μl methanol, 100 μl 1‐octanol, and pH 11 were applied as optimal values.

### 3.2 Method validation

Dynamic linear range (DLR), recovery, repeatability (RSD), enrichment factor (EF), limit of detection (LOD), and limit of quantification (LOQ) as details of merit were calculated under optimum situation (Table 1).

**Table 1:** Details of merit of the offered method for determination of four types of HAAs in doner kebab

Analyte	DLR (ng/g)	*R* ^2^	LOD (ng/g)	LOQ (ng/g)	RSD%	Recovery	EF
PhIP	1–1000	*R* ^2^ > 0.987	4.9	16.2	7.4	95	115
IQ	1–1000	*R* ^2^ > 0.987	5.3	17.5	8.1	89	120
MeIQ	1–1000	*R* ^2^ > 0.987	5.1	16.8	6.5	90	105
MeIQx	1–1000	*R* ^2^ > 0.987	4.8	15.8	8.3	97	110

Appropriate linearity was achieved in range 1–1,000 ng/g for HAA concentration with *R* ^2^ greater than 0.987. Estimating of repeatability was carried out by calculating the peak area of 6 same repeated experiments at the optimal condition and was considered as relative standard deviation (RSD %). RSD % was reported between 6.5% and 8.3% for HAAs. The recovery of the extraction process was determined by comparison of analyte amount before spiking, and the concentration recovered after spiking for each mentioned analytes. The obtained recovery values were 97% for MeIQx, 95% For PhIP, 89% for IQ, and 90% for MeIQ.

The enrichment factor was calculated according to the ratio of the concentration of analytes in the extraction solvent after microextraction to concentration analyte in the primary solution before microextraction process. This factor was obtained between 105 and 120. The LOD and LOQ in the optimum condition of LDS/DLLME/RP/HPLC were 4.9 and 16.2 ng/g for PhIP, 5.3 and 17.5 ng/g for IQ, 5.1 and 16.8 ng/g for MeIQ, and 4.8 and 15.8 ng/g for MeIQx, respectively.

### 3.3 Comparison of the offered method with other methods

As mentioned in Table 2, our study method for the determination of HAAs in doner kebab samples has been comprised of other analytical techniques done in previous studies. All of these techniques tried to offer suitable sample preparations which can remove food matrices interferences and extract HAAs from several heated food samples, for example, soup cubes, and meat extracts. Liquid chromatography has been employed as a common analytical instrument. HPLC coupled with the different detector, especially MS detector, showed the acceptable and nice results to quantify HAAs in food samples.

**Table 2:** Comparison between proposed method and other methods for determination of HAAs

Method	Samples	Recovery (%)	Linear range (ng/g)	LOD (ng/g)	RSD (%)	*R* ^2^	EF	References
QuEChERS‐UHPLC‐MS/MS	Coffee products	81.6–100	–	–	–	>0.996	–	Yanping et al. (2019)
SPE‐IPC‐CEAD^a^	Soup cubes	54.8–100	5.25–176	0.1–1.1	3–5.8	–	–	Krach and Sontag (2000)
SPME‐HPLC^b^	Food samples	64–112	0.4–400	0.3–14	1.3–22	0.945–0.999	‐	Cárdenes et al. (2004)
SPE‐HPLC‐FLD^c^	Roasted coffee	40–56	–	0.21–0.51	–	–	–	Karpavičiūtė et al. (2017)
PLE‐LC‐MS/MS^d^	Meat extracts	45–79	–	0.02–1	13 > RSD	0.945–0.999	–	Khan et al. (2008)
MAE‐LDS‐DLLME‐RP‐HPLC^e^	Doner kebab	89–97	1–1000	4.8–5.1	6.5–8.3	>0.987	105–120	This work

a Solid‐phase extraction/ion‐pair chromatography with coulometric electrode array detection

b Solid‐phase microextraction/high‐performance liquid chromatography

c Solid‐phase extraction/high‐performance liquid chromatography with fluorescence detector

d Pressurized liquid extraction/liquid chromatography–tandem mass spectrometry

e Microwave‐assisted extraction/ dispersive liquid–liquid microextraction/ high‐performance liquid chromatography

All sample preparation, including conventional (SPE and PLE) and microextraction methods (SPME and DLLME), presented efficient extraction of a target analyte in food matrices. All variables presented in Table 2 showed a good dynamic linear range, and our method has a wide range of linearity from 1 to 1,000 ng/g compared to other methods. Extraction recoveries for all methods can be considered as acceptable, while the lowest recovery was obtained as 89% while compared to 45, 40, 64, and 54% as the corresponded values for other methods. The LODs for SPME and also MS techniques were acceptable because of free solvent extraction and powerful detection, respectively. LOD for the offered method is acceptable. Repeatability for SPE and SPME methods was reported 3 to 5.5% and 1.3 to 22%. PLE reference was reported RSD lower than 13%.

These results confirm that the type of food matrices has significantly affected the determination of HAA levels. Other details of merits confirm the effect of food type on measuring of HAAs in different heated food samples again. Table 2 has presented the results of the suggested technique and other analytical techniques employed to quantify HAAs in heated meat samples.

### 3.4 Applicability of applied technique on different doner samples

**Figure 3:**
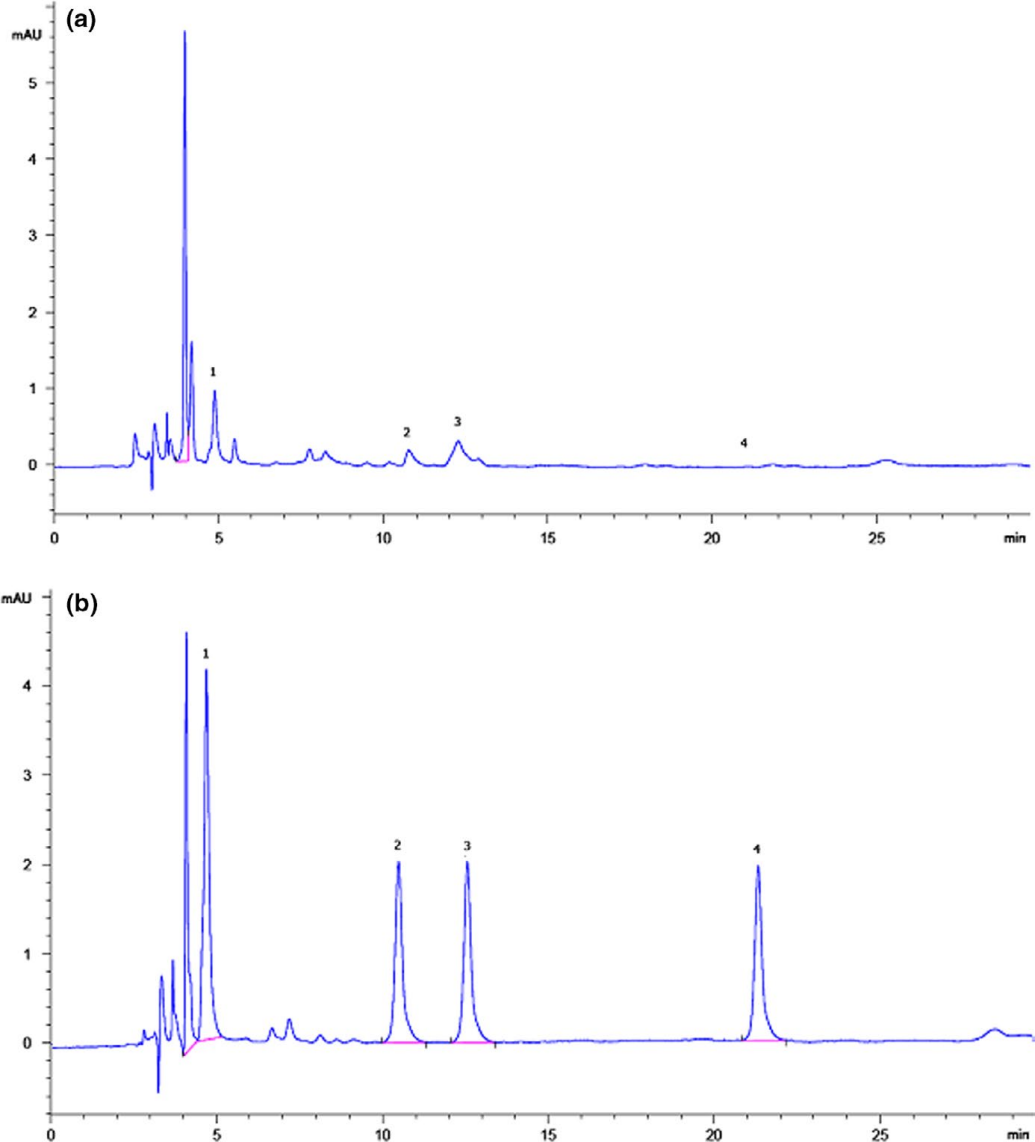
The chromatogram obtained by MAE/LDS/DLLME/RP/HPLC for sample (5) under optimum conditions: nonspiked and spiked peaks with 100 ng/g of four HAAs. PhIP (1), IQ (2), MeIQ (3), MeIQx (4)

Due to the evaluation of the suggested method, we purchased five doner kebab samples from five different food restaurants (Tehran, Iran) and applied the method to analyze HAAs (Table 3). Figure 3 indicates that chromatograms established using MAE/LDS/DLLME/HPLC for doner kebab samples 5; (a) before and (b) after spiking by HAA standard at three level concentration (10, 50 and 100 ng/g). This chromatogram did not have any sample tissue interferences. The results of this experiment demonstrated that sample 2 contained a high concentration of MeIQx among the other samples. According to Table 3, the lowest and highest level of HAAs is depicted 8.90 ng/g and not detected for PhIP, 15 ng/g and not detected for IQ, 20.50 ng/g and not detected for MeIQ, and 25.20 ng/g and not detected for MeIQx, respectively. Also, sample 5 contains the highest level of ∑4HAAs (56.80 ng/g), and sample 3 has the lowest level of ∑4HAAs (25.30 ng/g) among the other samples. It could explain that sample 2 may have more level of lean meat or has been received more time of frying. Sample 3 may be prepared with more spices which decreased the HAA formation or detoxified these toxicants from doner samples. Some other factors, like a low amount of lean meat or light heating procedure, could be considered as the possible reasons for the low formation of HAAs in these samples. According to Table 3, the most and the least total mean values of HAAs in doner kebabs were calculated 13.30 ng/g for MeIQx and 5.0 ng/g for IQ, respectively.

**Table 3:** Analytical results of HAAs in doner kebab samples by offered method

Samples	PhIP			IQ			MeIQ			MeIQx			∑4HAAs (ng/g)
	Concentration (ng/g)	Added amount (ng/g)	Analyzed amount (ng/g)	Concentration (ng/g)	Added amount (ng/g)	Analyzed amount (ng/g)	Concentration (ng/g)	Added amount (ng/g)	Analyzed amount (ng/g)	Concentration (ng/g)	Added amount (ng/g)	Analyzed amount (ng/g)	
1	8.9 ± 0.65^a^	10	17.95.±1.32	ND	10	8.5 ± 0.72	18.2 ± 5.39	10	25.38 ± 1.64	17.8 ± 20.25	10	17.26 ± 1.43	
		50	55.95 ± 4.14		50	44.50 ± 3.60		50	61.38 ± 3.98		50	56.06 ± 4.65	
		100	103.45 ± 7.65		100	89 ± 7.20		100	106.38 ± 6.91		100	114.26 ± 9.48	44.90
2	3.2 ± 0.23	10	12.54 ± 0.92	ND	10	8.5 ± 0.72	20.5 ± 4.57	10	27.45 ± 1.78	25.2 ± 4.61	10	34.14 ± 2.83	
		50	50.54 ± 3.73		50	44.50 ± 3.60		50	63.45 ± 4.12		50	72.94 ± 6.05	48.90
		100	98.04 ± 7.25		100	89 ± 7.20		100	108.45 ± 7.04		100	121.40 ± 10.07	
3	ND	10	9.5 ± 0.70	ND	10	8.5 ± 0.72	11.7 ± 30.51	10	19.53 ± 1.26	13.6 ± 3.24	10	22.89 ± 1.90	
		50	47.50 ± 3.51		50	44.50 ± 3.60		50	55.53 ± 3.60		50	61.69 ± 5.12	25.30
		100	95 ± 7.03		100	89 ± 7.20		100	100.53 ± 6.53		100	110.19 ± 9.14	
4	7.4 ± 0.54	10	16.53 ± 1.22	10.0 ± 45	10	17.80 ± 1.44	ND	10	9 ± 0.58	9.9 ± 2.91	10	19.30 ± 1.60	
		50	54.53 ± 4.03		50	53.40 ± 4.32		50	45.00 ± 2.92		50	58.10 ± 4.82	27.30
		100	102.03 ± 7.55		100	97.90 ± 7.92		100	90 ± 5.85		100	106.60 ± 8.84	
5	25.8 ± 1.90	10	34.01 ± 2.51	15.0 ± 1.5	10	22.25 ± 1.80	16.0 ± 9.15	10	23.40 ± 1.52	ND	10	9.7 ± 0.80	
		50	72.01 ± 5.32		50	57.85 ± 4.68		50	59.40 ± 3.86		50	48.50 ± 4.02	56.8
		100	119.51 ± 8.84		100	102.35 ± 8.29		100	104.40 ± 6.78		100	97 ± 8.05	
Total mean value ofHAA	11.32			5.00			13.28			13.30			–

a Mean value ± *SD*.

## 4 CONCLUSION

In the present work, doner kebab as a tasty food has been considered to investigate HAAs as toxic compounds formed into this food during heat processing. MAE/LDS/DLLME/HPLC has been applied as a quick, easy, selective, and sensitive technique for the extraction of four important types of HAAs including PhIP, IQ, MeIQ, MeIQx from doner kebab. The optimizations of the main parameters in the extraction step been carried out using CCD. In optimal condition, the exact amount of HAAs has been determined. The proposed method could significantly remove the interferences of food matrices, and the analysis procedure has been done in a short time with high recovery. ∑4HAAs in doner kebab samples have been detected lower than 50 ng/g. The comparison of our method with other methods showed that the offered method has high capability to determine HAAs in food samples with high extraction efficiency. Type and distance of the heating source, the amount of fat, and amount of spices can be considered as other affected parameters to investigate the amount of HAAs in doner kebab.

## CONFLICTS OF INTEREST

The authors declare that they have no competing interests.

## ETHICAL APPROVAL

The human and animal testing was unnecessary in the current study.
